# Nurses´ perceptions of automated dispensing cabinets – an observational study and an online survey

**DOI:** 10.1186/s12912-020-00420-2

**Published:** 2020-04-19

**Authors:** Riikka Metsämuuronen, Hannu Kokki, Toivo Naaranlahti, Minna Kurttila, Reeta Heikkilä

**Affiliations:** 1 School of Pharmacy, University of Eastern Finland, PO Box 1627, FI-70211 Kuopio, Finland; 2 School of Medicine, University of Eastern Finland, PO Box 100, FI-70029 Kuopio, KYS Finland; 3 School of Pharmacy, University of Eastern Finland, Kuopio, Luolatie 22, FI-70780 Kuopio, Finland; 4 Kuopio University Hospital Pharmacy, Kuopio, Kelkkailijantie 3, FI-70200 Kuopio, Finland; 5 Doctor of Pharmacy, Lecturer, School of Pharmacy, University of Eastern Finland, Kuopio, PO Box 1627, FI-70211 Kuopio, Finland

**Keywords:** Automated dispensing cabinet, ADC, Hospital pharmacy, Drug storage and distribution system, Observation, Survey, Nurse, Perception, Work efficiency

## Abstract

**Background:**

Thirty-two automated dispensing cabinets (ADCs) were introduced in May 2015 in Kuopio University Hospital, Finland. These medication distribution systems represent relatively new technology in Europe and are aimed at rationalising the medication process and improving patient safety. Nurses are the end-users of ADCs, and it is therefore important to survey their perceptions of ADCs. Our aim was to investigate nurses’ perceptions of ADCs and the impacts of ADCs on nurses’ work.

**Methods:**

The study was conducted in the Anaesthesia and Surgical Unit (OR) and Intensive Care Unit (ICU), of a tertiary care hospital, in Finland. We used two different research methods: observation and a survey. The observational study consisted of two 5-day observation periods in both units, one before (2014) and the other after (2016) the introduction of ADCs. An online questionnaire was distributed to 346 nurses in April 2017. The data were analysed using descriptive statistics including frequencies and percentages and the Chi-Square test.

**Results:**

The majority (*n* = 68) of the 81 respondents were satisfied with ADCs. Attitudes to ADCs were more positive in the ICU than in the OR. Nearly 80% of the nurses in the ICU and 42% in the OR found that ADCs make their work easier. The observational study revealed that in the OR, time spent on dispensing and preparing medications decreased on average by 32 min per 8-h shift and more time was spent on direct patient care activities. The need to collect medicines from outside the operating theatre during an operation was less after the introduction of ADCs than before that. Some resistance to change was observed in the OR in the form of non-compliance with some instructions; nurses took medicines from ADCs when someone else was logged in and the barcode was not always used. The results of the survey support these findings.

**Conclusions:**

Overall, nurses were satisfied with ADCs and stated that they make their work easier. In the ICU, nurses were more satisfied with ADCs and complied with the instructions better than the nurses in the OR. One reason for that can be the more extensive pilot period in the ICU.

## Impact and implications of findings on practice

- Automated dispensing cabinets (ADCs) can facilitate nurses´ work and improve work efficiency.
- When hospitals and other health care facilities are planning to develop and reorganise the medication process, the introduction of ADCs is worth considering.
- The successful introduction of ADCs requires commitment and acceptance from employees, and resistance to change can restrict the system from working optimally.
- A pilot phase can reveal opportunities to improve the process and benefit to manage the change.
- Attitudes to ADCs were mainly positive among nurses in Kuopio University Hospital, and the introduction of the ADC system succeeded rather well.

## Background

Automated dispensing cabinets, ADCs, were introduced in the 1980s in the United States, since which time they have been increasingly used to automate and rationalise the medication process in hospitals and other health care facilities [1]. These medication storage systems decentralise the distribution of drugs near the patient and provide quick access to medicines for the nurse. ADCs have the potential to reduce medication errors and to improve the work efficiency of pharmacy and nursing staff [1–5]. Barcode scanning, generally combined with ADCs, reduces the risk of errors both when drugs are removed from an ADC and when they are being placed in the cabinet. ADCs can also help to control illegal drug delivery for other than patient use. Furthermore, ADCs can help to account for medicine, billing and inventory management.

Kuopio University Hospital (KUH), Kuopio, Finland, a tertiary care hospital, has a catchment of 250,000 people, 90,000 annual patients and 20,000 annual operations. In May 2015, a new part of the hospital was completed and 32 ADCs (eMED ICON, NewIcon, Kuopio, Finland) were introduced there in the Operating Rooms of the Anaesthesia and Surgical Unit (OR), and the Intensive Care Unit (ICU). Most of KUH’s ADCs are placed into the wall structures of operating theatres and patient rooms using an innovative pass-through method [6]. This system allows the ADCs to be filled from outside the room and the medicines obtained inside the room. This is expected to reduce unnecessary movement into and out of the operating theatre during operations. Each ward has also a central ADC in the medication room, in which drugs that are seldom needed are stored.

Before collecting a medicine from the ADC, a user logs into the system. Light guidance helps to find the selected product. Each medicine package taken from a cabinet is documented by scanning its barcode to ensure that the right medicine has been taken. The system manages storage control and provides computer-controlled, real-time monitoring and tracking of medicine use and waste. Since the introduction of ADCs in KUH, the billing system has changed; nowadays wards pay only for the medicines they have used, and the medicines are owned by a pharmacy until they are removed from the ADC. The ADCs are restocked by pharmacy technicians.

Before introducing the ADCs in clinical use, the nursing staff could practise their use during several pilot phases. The idea of the pilot phases was to gather user experience, recognise problems and improve the usability of the ADCs. The pilot phase lasted 5 months in the OR and 8 months in the ICU. The most important change of ADCs, made based on the pilot phase, was the pass-through principle that allows nurses to use the ADC without disturbing the patient care activities.

Change management has an essential role in introducing ADC systems because the introduction of new systems causes often resistance to change [7]. The major challenges in introducing such systems successfully are usually more behavioural than technical. Individuals have to give up their familiar routines and invest their time and energy in learning the new system. The successful introduction of ADCs requires commitment and acceptance from employees, and resistance to change can restrict the system from working optimally.

Nurses are the end-users of ADCs, and their perceptions of ADCs are therefore important to survey. The aim of our study was to assess nurses´ perceptions of ADCs and the impacts of ADCs on nurses’ work. Our study hypothesis was that implementation of ADCs would decrease nurses’ time spent on dispensing and preparing medicines and that ADCs would be accepted smoothly by employees after pilot phases.

## Methods

We conducted the study in KUH using direct observation of nurses both before (2014) and after (2016) the introduction of ADCs together with an online survey for nurses in 2017.

### Observation

The first of the two observation periods was carried out in April–May 2014, a year before the introduction of ADCs, in the OR and ICU. The second observation period was conducted in the same units in April–May 2016, a year after the introduction of ADCs. The observation periods consisted of five consecutive days (8 hours per day) in both units.

The observation data were collected by the same observer (RM) both before and after the introduction of ADCs. She observed a different person on each day during each 8-h shift. The activities of the nurses, the time spent with medication-related and other tasks, and movements into and out of the operating theatre during surgical procedures were recorded. Medication-related activities included searching for medicines, taking medicines from the cabinet and preparing and administering medicines, while other activities included monitoring patients, recording patient data, stock managing and personnel traffic in the unit. The time spent by nurses on different activities was assessed by measuring the duration of each activity. Any signs of resistance to change among nurses were recorded. A pilot data collection period was used before the actual observation period. All observations were first recorded manually, and at the end of the observation day entered into an electronic database.

For the analysis, the observation data from 2014 and 2016 were compared with each other. Firstly, we analysed how much time nurses spent on different tasks and whether the ADCs had any impact on time management and workflow. In the OR, we investigated how many times nurses left the operating theatre during an operation to collect medicines from outside the room.

### Online survey

An online questionnaire was developed for this study and it was distributed in April 2017 to 346 nurses of the OR and ICU (See Appendix 1). A link to the questionnaire on Surveypal (Surveypal Inc., Tampere, Finland) was sent to the nurses through their official work e-mails with a 4-week deadline for responses.

The questionnaire was piloted on five nurses. They checked whether the questions were understandable, appropriate, logical, non-confusing and non-leading. Their answers were included in the final survey.

The questionnaire consisted of different parts: the nurses´ sociodemographic and practice characteristics, closed- and open-ended questions about whether the ADCs have had an impact on their work and whether they have had problems with ADCs, questions about the use of the ADCs, 18 statements on the ADCs with a 5-point Likert scale (1 = strongly disagree, 5 = strongly agree), questions about the impacts of ADCs on patient safety, a question about overall satisfaction and two open-ended questions about suggestions for improvements and free comments. Nurses working in the OR were also asked whether the ADCs increased or decreased the need to collect medicines from outside the operating theatre during an operation.

The online survey data were exported direct from the Surveypal program to IBM SPSS Statistics for Windows, Version 23.0. (International Business Machines Corporation, Armonk, NY, USA). The data were analysed using descriptive statistics including frequencies and percentages and the Chi-Square test. We combined the strongly disagree and disagree, and the strongly agree and agree, answers to present the data concisely.

### Ethics approval

Ethics approval for the study protocol was obtained from the Research Ethics Committee of the Hospital District of Northern Savo, Kuopio, Finland (152/2016, April 5, 2016). The study had institutional approval. Informed verbal consent was obtained from each nurse prior to the observations. Participation in the survey was voluntary and responses were gathered anonymously.

## Results

### Observation study

#### Observations in the Anaesthesia and surgical unit

##### Nurses’ use of time

The time spent on dispensing and preparing medications, including searching for medicines, taking medicines from the cabinet, preparing the medicines and marking them, in the operating theatre for elective surgery decreased on average by 32 min per 8-h shift after the introduction of ADCs; in 2014 nurses spent on average 55 min, and in 2016 on average 23 min, on these activities per 8-h shift. The time saved, 32 min, was spent on patient management and monitoring in the operating theatre; in 2014 nurses spent on average 243 min, and in 2016 on average 272 min, per 8-h shift on patient care in the operating theatre during an operation, an increase of 29 min per 8-h shift. Time spent on other duties was the same between 2014 and 2016.

##### Movement of nurses during an operation

The need to collect medicines from outside the operating theatre during an operation was less in 2016 than in 2014. In 2014, the nurses under observation collected the medicines or solutions from outside the operating theatre during the operation in seven out of eleven (64%) operations observed, whereas in 2016 the figure was in only one operation out of eight (13%). In 2014, there were altogether 15 collections compared to three in 2016. In 2014, there were up to six collections during a single operation.

##### Resistance to change

Some resistance to change was observed in the OR, where a few deficiencies were noted in documenting the drugs removed from the ADC and the use of barcodes. Some nurses took medicines from the ADC without recording the fact, which caused errors in the stock balance. Neither did all nurses use the barcode identification. In some cases, nurses also took medicines from the ADC when someone else was logged in.

#### Observations in the Intensive care unit

##### Nurses’ use of time

In the ICU, nurses´ time spent on different tasks was similar between 2014 and 2016. In 2014, nurses spent on average 78%, and in 2016 80% of their working time in a patient room treating and monitoring patients.

##### Resistance to change

Nurses´ perceptions of ADCs were more positive in the ICU than in the OR. Nurses logged in and out correctly and, with only a few exceptions, did not use the ADC when someone else was logged in. They also documented the removal of a medicine in the data system by scanning the barcode.

### Online survey

The response rate of the survey was 23% (81/346). The nurses´ sociodemographic and practice characteristics are presented in Table 1.

**Table 1:** Nurses´ sociodemographic and practice characteristics (*n* = 81)

Characteristics	Number (%)
**Title**	
Staff nurse	79 (98)
Head nurse	2 (2)
**Nursing unit**	
Anaesthesia and Surgical Unit	52 (64)
Intensive Care Unit	29 (36)
**Sex**	
Female	67 (83)
Male	14 (17)
**Age (years)**	
20–29	10 (12)
30–39	21 (26)
40–49	23 (28)
50–59	26 (32)
60 or over	1 (1)
**Years of work experience in the current unit**	
< 1	5 (6)
1–4	24 (30)
5–10	15 (19)
11–15	8 (10)
> 15	25 (31)
Unknown	4 (5)
**Did you work in your current unit before the ADC system?**	
Yes	65 (80)
No	16 (20)
**How often do you use an Automated Dispensing Cabinet?**	
Every workday	75 (93)
Weekly but not every workday	4 (5)
Less than weekly	2 (2)

#### Nurses´ perceptions

A high proportion of the nurses (84%, 68/81) were satisfied with the ADCs (Table 2). Most nurses (81%, 66/81) found ADCs easy to use and 85% (69/81) agreed that the concept of ADCs is good (Table 3). The majority of the respondents (75%, 61/81) disagreed with the statement that they would rather return to the old stock system, while most of the nurses (74%, 60/81) also disagreed that the process of patient medication had become more difficult.

**Table 2:** Overall satisfaction with the Automated Dispensing Cabinets (ADC), impacts of ADCs on nurses’ work and patient safety and prevalence of problems with ADCs (*n* = 81)

Question	Anaesthesia and Surgical Unit (*n* = 52) Number (%)	Intensive Care Unit (*n* = 29) Number (%)
**How satisfied are you with ADCs overall?**		
Satisfied	42 (81)	26 (90)
Neither satisfied nor dissatisfied	8 (15)	3 (10)
Dissatisfied	2 (4)	0 (0)
**Have ADCs had an impact on your work?**		
Yes, they have made my work easier.	22 (42)	23 (79)
Yes, partly they have made my work easier and partly more difficult.	17 (33)	5 (17)
Yes, they have made my work more difficult.	3 (6)	0 (0)
No, they have not made my work easier or more difficult.	10 (19)	1 (3)
**Have you had problems with ADCs?**		
Yes, daily	5 (10)	0 (0)
Yes, weekly	18 (35)	8 (28)
Yes, monthly	12 (23)	15 (52)
Yes, less than monthly	14 (27)	5 (17)
No, I have not	3 (6)	1 (3)
**How do ADCs affect patient safety?**		
ADCs improve patient safety.	30 (58)	20 (69)
ADCs partly improve and partly adversely affect patient safety.	10 (19)	4 (14)
ADCs weaken patient safety.	0 (0)	0 (0)
ADCs have no effect on patient safety.	12 (23)	5 (17)

**Table 3:** Perceptions of the nurses in the Anaesthesia and Surgical Unit (OR, n = 52) and Intensive Care Unit (ICU, n = 29) about the Automated Dispensing Cabinet (ADC) system

Statement	Disagree, %		Neutral, %		Agree, %	
	OR	ICU	OR	ICU	OR	ICU
The log-in and identification to access the ADC are time-consuming	39	62	25	17	37	21
Medicines are easy to find in the ADC.	15	17	12	0	73	83
I often have to wait to access the ADC while another user accesses it.	69	55	14	24	17	21
It occurs daily in our unit that nurses take medicines from the ADC when someone else is logged in.	4	31	2	21	94	48
ADCs are easy to use.	12	3	13	3	75	93
Some necessary medicines are missing from the ADC daily.	73	66	13	17	13	17
It is common in our unit that the medicines removed from the ADC are not always documented in the system.	35	69	40	21	25	10
I now spend less time ordering and preparing medicines than before the ADC system was installed.	15	10	37	31	48	59
Pass-through ADCs reduce unnecessary movement into and out of the operating theatre and patient rooms.	10	3	12	3	79	93
Adequate training is given on how to use the ADC.	2	0	13	14	85	86
The restocking service offered by the Pharmacy has worked well.	17	14	15	14	67	72
ADCs reduce medication selection errors.	13	7	40	28	46	66
Neglecting to record the removal of a medicine poses a risk to patient safety.	23	21	19	17	58	62
Using a barcode when taking medicines from the ADC improves patient safety.	13	10	29	10	58	79
The concept of ADCs is good.	4	0	13	10	83	90
I would rather return to the old stock system.	71	83	15	10	13	7
The process of patient medication has become more difficult.	67	86	23	7	10	7
ADCs reduce the risk of medication misuse by staff.	31	21	29	17	40	62

In the ICU, a higher proportion of the nurses (79%, 23/29) said that the ADCs have made their work easier compared to the nurses in the OR (42%, 22/52) (Table 2). Over half (59%, 17/29) of the nurses in the ICU and 48% (25/52) in the OR found that with the ADCs they spend less time ordering and preparing medications than before the ADC system was installed. In the open-ended answers, nurses mentioned most frequently that the fact that medicines are easier to find in the ADC and that they are easily available near the patient makes their work easier. The capability of ADCs to show where the needed medicine is, if the ADC in question does not contain it, was mentioned as one factor that makes nurses´ work easier. Also seen as beneficial were the use of a label printer and the restocking service offered by the Pharmacy.

A high proportion of the respondents (95%, 77/81) reported problems with ADCs, although only five (6%) had had problems daily (Table 2). Over a third (37%, 19/52) of the nurses in the OR and 21% (6/29) in the ICU felt that the log-in and identification needed to access the ADC are time-consuming (Table 3). The most frequently mentioned problems were system failures and other technical problems such as the problems with logging in, door opening and label printers. Stock balance errors and the occasional lack of some medicines were also experienced as problems.

Nearly two-thirds (62%, 50/81) of the nurses felt that ADCs improve patient safety (Table 2). One out of six (17%, 14/81) believed that ADCs have both positive and negative impacts on patient safety. In the ICU, 66% (19/29) and in the OR, 46% (24/52) of the respondents agreed that ADCs reduce medication selection errors. Features of ADCs that improve patient safety, as most frequently mentioned in the open-ended answers, were barcode scanning, greater confidence that the right medicine has been taken, and the informative medicine labels which the ADC automatically prints. Light guidance was also mentioned as one of the factors that improve patient safety as it helps to find the product. Factors mentioned as adversely affecting patient safety were technical problems with ADCs and stock balance errors caused by neglecting to record the removal of a medicine.

#### The use of automated dispensing cabinets

The results of the online survey support the observation that in the OR ADCs are often used when someone else is logged in (Table 4). One-third (37%, 19/52) of the respondents in the OR stated that they are logged in only in 0–2 cases out of ten with their own identification when they use the ADC. In the ICU, the figure was 0% (0/29).

**Table 4:** Use of Automated Dispensing Cabinets (ADCs) with the user’s own identification, use of a barcode and recording the removal of a medicine in the Anaesthesia and Surgical Unit (OR, *n* = 52) and Intensive Care Unit (ICU, *n* = 29). Data are number of cases (percentage)

In how many cases out of ten	Number of cases out of ten							
	0–2		3–5		6–8		9–10	
	OR	ICU	OR	ICU	OR	ICU	OR	ICU
You are logged in with your own identification when you use the ADC?	19 (37)	0 (0)	10 (19)	0 (0)	3 (6)	8 (28)	20 (38)	21 (72)
You use the barcode when you take a medicine from the ADC?	25 (48)	3 (10)	9 (17)	4 (14)	13 (25)	14 (48)	5 (10)	8 (28)
You record the removal of a medicine?	0 (0)	0 (0)	0 (0)	0 (0)	3 (6)	1 (3)	49 (94)	28 (97)

Almost all (94%, 49/52) nurses in the OR agreed also with the statement that it is a daily occurrence in their unit for nurses to take medicines from the ADC when someone else is logged in. The corresponding figure was 48% (14/29) in the ICU.

The results of the observation and the survey are similar in relation to the use of a barcode also. Nearly half of the respondents in the OR (48%, 25/52) stated that they use the barcode only in 0–2 cases out of ten when they take a medicine from the ADC (Table 4). The corresponding figure was 10% (3/29) in the ICU. All products do not have barcodes which can partly explain the non-use of barcodes. However, as the observation study revealed, a barcode was not always used even when one was available. Even though a barcode is not always used, 65% (53/81) felt that the use of a barcode, when medicines are taken from the ADC, improves patient safety (Table 3).

Almost all (95%, 77/81) of the respondents stated that they record the removal of a medicine in 9–10 out of ten cases. There were no differences between the two units. However, 20% (16/81) of the nurses agreed that in their unit medicines removed from the ADC are not always documented to the system (Table 3). More than half (59%, 48/81) agreed that neglecting to record the removal of a medicine poses a risk to patient safety.

## Discussion

Time spent on dispensing and preparing medications decreased on average by half an hour per 8-h shift in the OR. The time saved was used on patient management and monitoring in the operating theatre during operations. The introduction of ADCs may have contributed to the faster collection of medicines as all the necessary medicines and associated supplies have been stocked in the ADCs of the operating theatres being available near the bedside. If some medicines lack in the ADC, nurses can collect them from the central ADC of the unit instead of collecting them from peripheral storage points, which reduces the number of steps taken by nursing staff. Especially in the OR standardised medications and well-organised storage of medicines in the unit are important principles in improving fluent workflow and safe medication process if installing the ADCs is not possible [8]. The surface area of the former ICU was remarkably smaller compared to the new unit, and the distances required for medicine and equipment collection were short, which may explain why the time spent on dispensing and preparing medications did not decrease in the ICU. One explaining factor for the difference between the OR and ICU could be also the presence of a whole-time ward pharmacist in the ICU before and after the installing the ADCs. In the OR, the pharmacy service has been part-time and mainly technical. The nurses in the OR were more involved in the pharmaceutical tasks (e.g. dispensing and reconstitution) compared to the situation in the ICU and thus, the ADCs streamlined their work more.

In general, the nurses were satisfied with ADCs and expressed they have made their work easier. This is consistent with the findings of Rochais et al. (2014) and Zaidan et al. (2016), who have investigated nurses´ perceptions of, and satisfaction with, the use of ADCs [9, 10].

Employees’ experiences in adapting to technological change were investigated by Sarnola et al. (2019) [11]. That study, which was carried out in KUH, revealed that work performance and efficiency-related (e.g. workflow, technology, training and ergonomics) positive factors enhanced employees’ ability and willingness to adapt to the change. Correspondingly negative factors which diminished employees’ ability to adapt were related to work performance but to individual-related factors (e.g. attitudes, knowledge and motivation) also. In this study, 90% of the respondents in the ICU were satisfied with the ADCs in general compared to 81% in the OR (Table 2). Nurses’ perceptions in the ICU were more positive than in the OR concerning the work performance related factors such as “easy to use” and “decreased movement” (Table 3). If nurses felt that the ADCs were beneficial, they adapted more easily to the change which could be seen in the ICU responses.

The introduction of new systems can cause resistance to change among staff at first, which can appear as non-compliance with instructions. In the present study the non-compliance with instructions was most apparent in the OR (Table 4). The observation established some deficiencies concerning the use of a barcode, and the results of the survey supported this finding.

The removal of a medicine was not always recorded, even though 95% of the nurses stated that they record it in 9–10 cases out of ten. The idea of recording is to ensure that the ADC always contains the appropriate quantities of medicines as the system manages stock control. It can be a risk to patient safety if an important product is lacking because someone has not recorded its removal. It also causes unnecessary work for pharmacy technicians. The problem is easily solved if everybody is motivated to comply with the instructions.

During the observation the nurses took medicines from the ADC when someone else was logged in, especially in the OR, and this was supported by the results of our survey. Work in an operating theatre is demanding, and the nurses have to scan all the time the patient, monitors, machines and infusions [8, 12]. In hectic situations when nurses are required to take medicines from cabinets immediately, they may consider logging in and recording time-consuming while their work is intensively focused to the patient monitoring. Based on the observations made in the present study, however, logging in the ADC does not take that much time, and in an emergency, logging can be overridden. Moreover, fewer than one-third of the respondents agreed that the log-in and identification process needed to access the ADC is time-consuming. This is supported by our measurements: the average time spent at the ADC on collecting a medicine is less than 16 s. One of the aims of the user recognition before medicine removal is to help resolve any unclear cases and to prevent medicine abuse by hospital staff [1, 13].

Trust is a significant factor in engaging employees in the management of change caused by automation [14, 15]. Reliable performance of the ADCs supports adaptive reliance on the new automation and the change is then considered trustworthy. The survey reveals (Tables 2 and 3) that the respondents in the ICU had a more positive attitude to the new automation meaning they had higher trust on it. In the OR, where the non-compliance and misuse of the ADCs were more prominent (Table 4), the respondents did not rely on practical performance of the ADCs that much which could cause a lack of confidence with the new technique. This can explain in the OR, why that many nurses did not comply with the instructions.

The reason why the nurses in the ICU were more satisfied with the ADCs and adapted better to the change than the nurses in the OR can be the difference in the extent of the pilot phase. In the ICU, the pilot period before the introduction of ADCs was more extensive and all nurses had the opportunity to practise the use of ADCs. The ICU nurses had also better possibilities to influence on the design and properties of the ADCs and to develop a new operational model. Not this kind of ample pilot phase was able to conduct in the OR.

Successful introduction of ADCs demands communication, support and the adequate training of users to a high standard [7]. In the present study, most of the respondents agreed that adequate training was given on how to use the ADC, which could explain nurses´ satisfaction. When the users are involved in the change from the very beginning, resistance to change can be managed and converted into commitment and enthusiasm.

The traffic into and out of the operating theatre decreased significantly. ADCs reduced the need to collect medicines or solutions from outside the operating theatre during an operation. This may improve patient safety; as the time used in collecting the products reduced, the time spent on patient monitoring and management increased. In addition, room traffic between the operating theatre and the corridor is thought to e.g. increase the risk of contaminants getting into the operating theatre [16].

Some previous studies indicate that ADCs reduce medication errors [3, 4, 17, 18]. In the present study a majority of the nurses were confident that the new system may improve patient safety. The most frequently mentioned features of ADCs that promote patient safety were barcode scanning, greater confidence that the right medicine has been taken, and the labels which the ADC automatically prints containing all necessary information about the medicine. The effects of ADCs on patient safety should be further evaluated. Based on the present study and earlier data [4, 19] ADCs can improve the medication process by bringing the medicines near the patients, offering real-time stock control and helping to ensure with barcode scanning that the right medicine has been retrieved. ADCs have also an essential role in building the closed loop medication system which allows the real-time documentation of medication and patient information [20, 21].

One of the main limitations of the present study was that the observation period was relatively short. Working days can differ a lot in terms of the number, length and challenge of operations. Thus, the comparison would have been more soundly based if there had been longer observation periods at both units. However, observation is time-consuming and demanding, and collecting a large amount of data is therefore challenging [22]. On the other hand, direct observation allows an objective view to be formed of the phenomenon being explored. According to Ampt et al. [23], observational work sampling is a more reliable method for obtaining an accurate reflection of the duties of nurses than self-reported work sampling [23]. An external observer can also notice any shortcomings more easily than persons who meet these phenomena daily.

Observational studies of this kind are subject to the Hawthorne effect, i.e. the presence of an observer may change the behaviour of the participants [23, 24]. Nurses may make greater efforts and act more carefully while being observed. On the other hand, they can be more nervous when they are aware, they are being observed. However, the observation was similar across the pre- and post-intervention periods, and thus the presence of an observer should have had only a minor impact on our study findings. This is supported by the study by Ampt et al. [23], in which feedback from nurses indicated that the presence of a researcher did not influence their behaviour that much.

The response rate of the survey was relatively low (23%) which is typical of this kind of survey [25]. Thus, the results may not necessarily represent the views of the entire study population. However, as over 80 nurses responded, we consider the results reliable. As nurses are the end-users of ADCs, their opinions and feedback are important, and this information can be utilised to develop ADC systems further.

The strength of our study is that the observation periods were timed both before and after the introduction of the ADCs, and that the observations were made in the same way and by the same observer at both times. The second observation period was timed 1 year after the introduction of ADCs to ensure the nurses had had enough time to familiarize themselves with the new technology and environment.

Our study focused on the nurses´ perceptions of ADCs and the effects of ADCs on nurses´ work and time management. In further studies, an investigation into economic impacts could provide important information regarding the costs and benefits of introducing ADCs. Also, the impacts of ADCs on patient safety could be examined by comparing medication error rates before and after the introduction of ADCs.

## Conclusions

In general, nurses were satisfied with the ADCs and did not want to return to the old stock system. The attitudes towards ADCs were more positive and the compliance with instructions was better in the ICU than in the OR which can be explained by the more extensive pilot phase in the ICU. The introduction of ADCs decreased the time spent on dispensing and preparing medications in the OR. Thanks to the more efficient supply of medicines attributable to the ADCs, nurses had more time to focus on direct patient care. Traffic into and out of the operating theatre decreased significantly as there was less need to collect medicines from outside the operating theatre during operations. Based on our study, the introduction of ADCs is worth considering when hospitals and other health care facilities are planning to develop and reorganise the medication process. It is important to involve the users in the change and consider the needs of the users when designing automation systems.

## Supplementary information

**Additional file 1.** Appendix 1 Questionnaire on Nurses´ Perceptions of Automated Dispensing Cabinets. This additional file includes the questionnaire that was distributed online in April 2017 to 346 nurses of the Anaesthesia and Surgical Unit and Intensive Care Unit of Kuopio University Hospital.

## Data Availability

The datasets used and analysed during the current study are available from the corresponding author on reasonable request.

## Abbreviations

ADC: Automated dispensing cabinet
KUH: Kuopio University Hospital
ICU: Intensive care unit
OR: Anaesthesia and surgical unit
