# Supplementary material for: Nurses´ perceptions of automated dispensing cabinets – an observational study and an online survey
Source: BMC Nurs. 2020 Apr 19;19:27. doi: 10.1186/s12912-020-00420-2 (PMC7168878; doi:10.1186/s12912-020-00420-2)
Supplement: Supplementary file 1 — Additional file 1. Appendix 1 Questionnaire on Nurses´ Perceptions of Automated Dispensing Cabinets. This additional file includes the questionnaire that was distributed online in April 2017 to 346 nurses of the Anaesthesia and Surgical Unit and Intensive Care Unit of Kuopio University Hospital. [file 12912_2020_420_MOESM1_ESM.docx]

**Appendix 1. Questionnaire on Nurses´ Perceptions of Automated Dispensing Cabinets**

The aim of this online survey is to investigate nurses’ perceptions of Automated Dispensing Cabinets (ADCs) introduced in Kuopio University Hospital in 2015.

Answers are gathered anonymously.

Thank you for your time!​

1. Gender

____Female ____Male

1. Age

___20–29 years ___30–39 years ___40–49 years ___50–59 years ___60 years or over

1. Profession

____Nurse ____Other, what?

1. In which unit are you currently working?

____Anaesthesia and Surgical Unit

____Intensive Care Unit

____Other, in which?

1. Work experience in the current unit

____Less than a year. How many months?

____More than a year. How many years?

1. Did you work in your current unit before the ADC system?

__­__Yes ____No

1. How often do you use ADCs?

____Every workday

____Weekly but not every workday (skip question 8)

____Less than weekly (skip question 8)

____I do not use ADCs (skip question 8)

### How many times per day do you use ADCs?

____On average 1–5 times per day

____On average 6–10 times per day

____On average 11–15 times per day

____On average over 15 times per day

1. Have ADCs had an impact on your work?

____Yes, they have made my work much easier.

____Yes, they have made my work a little easier.

­____No, they have not made my work easier or more difficult (skip question 10)

____Yes, partly they have made my work easier and partly more difficult.

____Yes, they have made my work a little more difficult.

____Yes, they have made my work much more difficult.

1. How have ADCs made your work easier or more difficult?

__________________________________________________________________________ ­­­­­­­­­­­­­­­­ __________________________________________________________________________

1. Have you had problems with ADCs?

____Yes, daily

____Yes, weekly

____Yes, monthly

____Yes, less than monthly

____No, I have not (skip question 12)

1. What kind of problems have you had with ADCs?

__________________________________________________________________________

__________________________________________________________________________

### Estimate, in how many cases out of ten you are logged in with your own identification when you use the ADC

Never Always

l l l l l l l l l l l

0 1 2 3 4 5 6 7 8 9 10

### Estimate, in how many cases out of ten you use the barcode when you take a medicine from the ADC

Never Always

l l l l l l l l l l l

0 1 2 3 4 5 6 7 8 9 10

### Estimate, in how many cases out of ten you record the removal of a medicine

Never Always

l l l l l l l l l l l

0 1 2 3 4 5 6 7 8 9 10

1. Do you agree with the following statements? Please, select the option that best describes your opinion. 1 = strongly disagree, 2 = somewhat disagree, 3 = neutral, 4 = somewhat agree, 5 = strongly agree.

| Statement | 1 | 2 | 3 | 4 | 5 |
| --- | --- | --- | --- | --- | --- |
| The log-in and identification to access the ADC are time-consuming | 1 | 2 | 3 | 4 | 5 |
| Medicines are easy to find in the ADC. | 1 | 2 | 3 | 4 | 5 |
| I often have to wait to access the ADC while another user accesses it. | 1 | 2 | 3 | 4 | 5 |
| It occurs daily in our unit that nurses take medicines from the ADC when someone else is logged in. | 1 | 2 | 3 | 4 | 5 |
| ADCs are easy to use. | 1 | 2 | 3 | 4 | 5 |
| Some necessary medicines are missing from the ADC daily. | 1 | 2 | 3 | 4 | 5 |
| It is common in our unit that the medicines removed from the ADC are not always documented in the system. | 1 | 2 | 3 | 4 | 5 |
| I now spend less time ordering and preparing medicines than before the ADC system was installed. | 1 | 2 | 3 | 4 | 5 |
| Pass-through ADCs reduce unnecessary movement into and out of the operating theatre and patient rooms. | 1 | 2 | 3 | 4 | 5 |
| Adequate training is given on how to use the ADC. | 1 | 2 | 3 | 4 | 5 |
| The restocking service offered by the Pharmacy has worked well. | 1 | 2 | 3 | 4 | 5 |
| ADCs reduce medication selection errors. | 1 | 2 | 3 | 4 | 5 |
| Neglecting to record the removal of a medicine poses a risk to patient safety. | 1 | 2 | 3 | 4 | 5 |
| Using a barcode when taking medicines from the ADC improves patient safety. | 1 | 2 | 3 | 4 | 5 |
| The concept of ADCs is good. | 1 | 2 | 3 | 4 | 5 |
| I would rather return to the old stock system. | 1 | 2 | 3 | 4 | 5 |
| The process of patient medication has become more difficult. | 1 | 2 | 3 | 4 | 5 |
| ADCs reduce the risk of medication misuse by staff. | 1 | 2 | 3 | 4 | 5 |

Patient safety

1. How do ADCs affect patient safety?

____ ADCs improve patient safety significantly (skip question 19)

____ ADCs improve patient safety a little (skip question 19)

____ ADCs have no effect on patient safety (skip question 18 and 19)

____ ADCs partly improve and partly adversely affect patient safety

____ ADCs weaken patient safety a little (skip question 18)

____ ADCs weaken patient safety significantly (skip question 18)

1. Which factors of ADCs improve patient safety?

__________________________________________________________________________

__________________________________________________________________________

1. Which factors of ADCs weaken patient safety?

__________________________________________________________________________­­

__________________________________________________________________________

1. How could ADCs be improved?

­­­­­­­­­­­__________________________________________________________________________

__________________________________________________________________________

­­­­­

1. How satisfied are you with ADCs overall?

___Completely dissatisfied

___Somewhat dissatisfied

___Neither satisfied nor dissatisfied

___Somewhat satisfied

___Completely satisfied

1. Free comments

­­­­­­­­­­­­­­­__________________________________________________________________________

__________________________________________________________________________

__________________________________________________________________________

Thank you for answering the questionnaire!
